# Sustainable Design and Wall Thickness Optimization for Enhanced Lifetime of Ultra-High Temperature Ceramic Matrix Composite Thruster for Use in Green Propulsion Systems

**DOI:** 10.3390/ma18133196

**Published:** 2025-07-07

**Authors:** Tamim Doozandeh, Prakhar Jindal, Jyoti Botchu

**Affiliations:** Space System Engineering, Faculty of Aerospace Engineering, Delft University of Technology, 2629 HS Delft, The Netherlands; tamimdoozandeh@gmail.com (T.D.); b.v.s.jyoti@tudelft.nl (J.B.)

**Keywords:** ultra-high temperature ceramic matrix composites, thruster design, green propulsion, finite element analysis, stress margin, failure–resilience assessments, transient, thermo-structural analysis

## Abstract

This study presents a comprehensive finite element investigation into the design optimization of an ultra-high temperature ceramic matrix composite thruster for green bipropellant systems. Focusing on ZrB_2_–SiC–Cfiber composites, it explores their thermal and mechanical response under realistic transient combustion conditions. Two geometries, a simplified and a complex full-featured model, were evaluated to assess the impact of geometric fidelity on stress prediction. The complex thruster model (CTM) offered improved resolution of temperature gradients and stress concentrations, especially near flange and convergent regions, and was adopted for optimization. A parametric study with nine wall thickness profiles identified a 2 mm tapered configuration in both convergent and divergent sections that minimized mass while maintaining structural integrity. This optimized profile reduced peak thermal stress and overall mass without compromising safety margins. Transient thermal and strain analyses showed that thermal stress dominates initially (≤3 s), while thermal strain becomes critical later due to stiffness degradation. Damage risk was evaluated using temperature-dependent stress margins at four critical locations. Time-dependent failure maps revealed throat degradation for short burns and flange cracking for longer durations. All analyses were conducted under hot-fire conditions without cooling. The validated methodology supports durable, lightweight nozzle designs for future green propulsion missions.

## 1. Introduction

The continuous pursuit of sustainable and durable propulsion systems for future space missions demands the development of high-performance materials and efficient structural designs. Ultra-high temperature ceramic matrix composites (UHTCMCs) offer a promising solution for high-temperature applications, such as upper-stage rocket thrusters, due to their excellent thermal stability, mechanical strength, and oxidation resistance. Optimizing structural elements, such as the nozzle wall thickness, can significantly enhance thruster lifetime, reduce system mass, and contribute to more environmentally sustainable space propulsion technologies. There is a significant rise in the deployment of small satellites, with nearly 95% of all spacecraft launched in 2022 being under 600 kg [1], and an increasing trend is anticipated for the future [2]. This growth necessitates the capability for frequent in-orbit maneuvers and space activities, where reliable propulsion systems play a critical role. Historically, chemical propulsion has been favored due to its high thrust-to-weight ratio, making it well-suited for in-space operations. However, traditional satellite propulsion systems have predominantly used highly toxic fuels such as hydrazine, posing environmental hazards and safety concerns during manufacturing, storage, and handling. To address these challenges, hydrogen peroxide (H_2_O_2_) has emerged as a greener propellant alternative, offering improved handling safety and reduced environmental impact [3,4,5]. Although the technology surrounding hydrogen peroxide propulsion is relatively immature compared to established systems, its adoption signals an important step toward sustainable space exploration.

During space operations, thrusters operate under extremely high temperatures and pressures, requiring materials with exceptional thermal shock resistance and mechanical stability. UHTCMCs, particularly those composed of ZrB_2_–SiC with short carbon fibers, exhibit very favorable physical properties for thruster manufacturing. Integrating advanced materials like UHTCMCs with green propellants presents a significant leap forward in sustainable, high-performance space propulsion technologies. This research aims to enhance propulsion systems by combining advanced material behavior analysis with propellant studies, focusing on improving both durability and performance to contribute meaningfully to the field of spaceflight mechanics. Currently, propulsion systems for upper-stage applications mainly use metallic alloys or conventional ceramic matrix composites to withstand thermal and mechanical loads. These materials, although reliable, often suffer from limitations such as lower maximum operating temperatures, higher mass, and vulnerability to oxidation and erosion [4]. UHTCMCs present an improvement by enabling operation up to 3000 K with thermal conductivities around 3.5 W/(m·K) [6], making them strong candidates for next-generation thruster designs. However, leveraging these material advantages fully requires thoughtful structural design, particularly in optimizing wall thickness to balance thermal protection, mechanical integrity, and system mass. Several previous numerical studies on traditional thruster materials analyzed loads and deflections based on ideal gas assumptions [7], while some emphasized the inclusion of temperature-dependent material properties and pressure–convection boundary conditions [6]. Some researchers even advocated for the integration of computational fluid dynamics (CFD)-derived loads into structural analysis frameworks to better understand the effects of thermal and pressure loads on structural deformation of the liquid bipropellant thruster [8]. A noticeable limitation in these studies is the assumption of steady-state operating conditions, often neglecting the critical startup and shutdown transients where thermal stresses can peak rapidly, particularly in high-temperature environments.

State-of-the-art studies on UHTCMC materials, such as those from the C3HARME project [9] have concentrated on material behavior characterization under extreme conditions but have not extended into integrated propulsion system design. Investigations by Zoli et al. [10] highlighted the critical role of the coefficient of thermal expansion (CTE) mismatch and its impact on microcrack formation under thermal shock conditions. Sciti et al. [11] further demonstrated that sharp convergent nozzle geometries exacerbate microcrack development under loading. However, while these studies identified vulnerabilities, they did not propose geometric or structural optimizations, such as thickness tapering, to mitigate damage. Thermal stress analyses on conventional materials indicated potential benefits from reducing wall thickness but stopped short of addressing this in the context of UHTCMC-based thrusters [9]. Challenges also persist in the accurate modeling of UHTCMC materials for finite element analysis. Although the CoPreD tool, developed during the C3HARME project [12], attempted to predict composite behavior, its limited accuracy, attributed to an insufficient understanding of microstructural influences, highlights the difficulty in creating reliable material models for simulation purposes. The lack of microstructural behavioral data makes it challenging to confidently specify material properties such as Young’s modulus, thermal expansion, and fracture strength, especially as these vary significantly with temperature and porosity. Further literature has documented how specific damage mechanisms manifest in UHTCMC components under operational stresses. A review discussing the mechanical behavior of carbon fiber reinforced TaC/SiC and ZrC/SiC composites identified the role of porosity and ceramic matrix strain in promoting crack growth at elevated temperatures [13]. These results indicate that controlling thermal gradients and stress distributions, particularly through geometric optimization, is crucial for extending the life of UHTCMC propulsion components. Specific studies on vibrational damage, ablation, and oxidation mechanisms revealed that long-fiber UHTCMCs outperform short-fiber variants under repeated thermal shocks and mechanical stresses [14,15]. Observations also indicated that oxidation could cause dimensional changes in nozzle throats, potentially impacting flow dynamics and structural integrity. The accumulation of oxidized layers under high heat flux conditions was found to alter the surface morphology, further emphasizing the need for careful structural and material design to preserve thruster performance over multiple firings [11].

In summary, the literature to date has advanced the understanding of UHTCMC material behavior but has not systematically explored how thruster wall thickness optimization can enhance operational lifetime, reduce thermal and mechanical stresses, and contribute to sustainability goals. Bridging this gap by integrating advanced material behavior knowledge with structural optimization under realistic operational conditions is the central aim of this study. The motivation for the present work stems from the growing need to develop lightweight, durable, and thermally resilient thrusters for green upper-stage propulsion systems using UHTCMC materials. A comprehensive parametric analysis of thruster wall thickness profiles is performed using finite element modeling, with thermal and mechanical loads derived from realistic hydrogen peroxide-based bi-propulsion conditions. The contribution of this work lies in providing detailed insights into how intelligent thickness tailoring can simultaneously enhance operational reliability and reduce environmental impact through mass optimization. The novelty is reflected in the coupling of advanced material modeling, sustainability-driven design, and real-world operational requirements, offering practical guidance for future thruster manufacturing and system design.

## 2. Materials and Methods

### 2.1. Materials and Geometry

The thruster in this study is fabricated entirely from an UHTCMC tailored to withstand the extreme thermal and mechanical conditions experienced during green bipropellant combustion. The material system comprises 65 vol% zirconium diboride (ZrB_2_), 20 vol% silicon carbide (SiC), and 35 vol% short carbon fibers. This composition is selected based on its proven capability to resist temperatures above 2500 K, its excellent oxidation resistance, and its enhanced fracture toughness imparted by fiber reinforcement [16].

To numerically model the temperature-dependent behavior of this UHTCMC, property estimation was carried out using classical micromechanics and rule-of-mixtures formulations. The upper and lower bounds for properties such as thermal conductivity (k), CTE, and specific heat (C_p_) were estimated using Equations (1)–(6).

For a property P,

(1) $$P_{upper}=V_{m}\;\cdot \;P_{m}+V_{f}\;\cdot \;P_{f}$$

(2) $$P_{lower}=\left(\frac{V_{v}}{P_{m}}+\frac{V_{f}}{P_{f}}\;\right)-1$$

where *P* denotes the material property that is being estimated, and *V* denotes the volume fraction. The subscripts *m* and *f* denote the matrix, which here is the UHTC, and fiber for the UHTCMCs, respectively.

The stiffness of the composite incorporating randomly oriented short fibers was derived from the orientation-averaged modulus expression as given in Equation (3)

(3) $$E=E_{m}\cdot \left(1-V_{f}\right)+V_{f}.\int [({cos}^{2}\theta -{sin}^{2}\theta )\;E_{f}{cos}^{2}\theta ]d\theta$$

with *E_m_* and *E_f_* denoting the matrix and fiber stiffness, respectively. *V_f_* denoting the fiber fraction, θ is the fiber orientation angle. This was further reduced to a simplified equation under an assumed Poisson ratio of 0.136 as shown in Equation (4):

(4) $$E=E_{m}-V_{f}\cdot \left(E_{m}-{0.562\;E}_{f}\right)$$

To account for porosity in the ceramic composite, a degradation factor was applied in Equation (4) leading us to a more specific Equation (5):

(5) $$E_{porous}=E_{0}\cdot \left(1-1.9P+0.9P^{2}\right)$$

where *E*_0_ is the modulus of the fully dense composite and *P* is the porosity volume fraction. Flexural strength was temperature-dependent and modeled based on Equation (6) as shown:

(6) $$\sigma_{f}\left(T\right)=\sigma_{f0}\cdot (1-V_{f})\cdot \left(1-\frac{0.562^{2}}{\pi }\right)\;$$

where $\sigma_{f0}$ is the room-temperature flexural strength of the fully dense composite. The resulting temperature-dependent material properties, obtained from Equations (1)–(6), implemented in the FEA simulation are listed in Table 1.

### 2.2. Geometry

The thruster studied in this work features a compact, upper-stage thruster profile intended for small satellite propulsion. The basic design was adapted from the Inconel thruster design received through non-academic collaboration from SolvGE. While acknowledging that individual design variations exist across different models and manufacturers, the selected dimensions are considered representative of standard practices within this class of thrusters, allowing for a robust demonstration of the thermo-structural analysis and optimization methodology. The profile includes a combustion chamber, throat (minimum diameter: 9 mm), and a divergent section. The total thruster length is 135.72 mm, with a chamber length of 60 mm and a nozzle length of 45.72 mm including a throat length of 6 mm.

**Figure 1:**
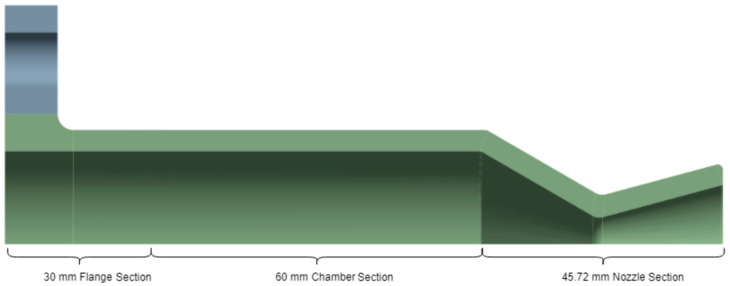
Sectional view of the basic thruster, highlighting the sections.

For structural analysis, the thruster was composed of three major zones, as illustrated in Figure 1. Zone A covers the flange section, where temperature and pressure buildup begin due to injection and combustion initialization, leading to thermal gradients. Zone B is the chamber section, where the flow develops and stabilizes, and the combustion is sustained. Zone C, the nozzle section, experiences the highest mechanical stress and steepest temperature differential due to gas acceleration. This segmentation enables a targeted application of meshing refinements and boundary conditions to capture localized behaviors.

Two distinct thruster profiles were developed to evaluate how geometric fidelity affects structural predictions. The first, termed the simple thruster model (STM), omits fillets, mounting flanges, and secondary contours to focus on primary geometry-stress interactions. The second, complex thruster model (CTM), incorporates all features and transition radii for a more realistic approximation of the manufactured part. This dual-model strategy allows us to balance computational cost against predictive accuracy, validate geometric simplifications, and capture stress peaks near complex curvature transitions, especially around the throat and flange roots.

A crucial component of this study is the exploration of thruster wall thickness as a design parameter. Nine configurations were analyzed based on overall uniformity, convergent and divergent thickness variations, and a linearly tapered profile reducing from 4 mm at the chamber interface to 2 mm at the nozzle exit. These cases are used to evaluate trade-offs between mass, stress resilience, and thermal behavior, and form the basis for structural optimization as discussed in Section 3.

### 2.3. Finite Element Analysis (FEA)

The finite element simulations were performed using ANSYS Mechanical 2023 R2. The analysis followed a stepwise procedure comprising geometry import, material property assignment, meshing and refinement, load/boundary condition application, and transient thermo-structural simulation.

#### 2.3.1. Boundary and Loading Conditions

Thermal and mechanical boundary conditions were defined based on the operational characteristics of the green bipropellant thruster using H_2_O_2_-kerosene. The internal surface of the nozzle was exposed to a time-dependent thermal profile peaking at 1643 K at the throat, derived from CFD simulations and assumed to follow the worst-case thermal load scenario. The outer wall was assigned a constant 300 K temperature to simulate space-like convective/radiative cooling. Pressure loads of 7.6 bar were applied uniformly to the inner wall, replicating the expected chamber operating pressure during peak thrusting. These loading conditions allow evaluation of the structural performance of the UHTCMC thruster under conservative, high-stress conditions, supporting lifetime and failure–resilience assessments.

The temperature distribution within the thruster is determined by solving the transient heat conduction equation, which can be generally expressed as Equation (7):

(7) $$\rho C_{p}\frac{\partial T}{\partial t}=\nabla \cdot (k\nabla T)$$

where ρ is the material density, *C_p_* is the specific heat capacity, *T* is temperature, *t* is time, and *k* is the thermal conductivity.

Stress calculations are based on linear elastic isotropic behavior, incorporating thermal strain effects. The governing constitutive Equation (8) for thermoelasticity is:

(8) $$\sigma_{ij}=C_{ijkl}\;(\varepsilon_{kl}-\alpha ∆T\delta_{kl})$$

where *σ_ij_* is Cauchy stress tensor, *C_ijkl_* is elasticity tensor, *ε_kl_* is total strain tensor, α is coefficient of thermal expansion (CTE) [K^−1^], Δ*T* is local temperature rise from the reference (stress-free) state, and *δ_kl_* is Kronecker delta.

The fundamental maximum principal stress (*σ*_1_) is derived from the Cauchy stress tensor using Equation (8). The principal stresses (*σ*_1_, *σ*_2_, *σ*_3_) are the eigenvalues of the Cauchy stress tensor, obtained by solving the characteristic Equation (9):

(9) $$\left|\begin{array}{ccc} \sigma_{x}-\sigma & \tau_{xy} & \tau_{xz}\\ \tau_{yx} & \sigma_{y}-\sigma & \tau_{yz}\\ \tau_{zx} & \tau_{zy} & \sigma_{z}-\sigma \end{array}\right|=0$$

where *σ* represents the principal stress values (*σ*_1_, *σ*_2_, *σ*_3_), and *σ_x_*, *σ_y_*, *σ_z_*, *τ_xy_*, *τ_yz_*, *τ_zx_* are the components of the stress tensor in Cartesian coordinates. This metric is particularly crucial for brittle materials like UHTCMCs, where failure is often initiated by tensile stress. The ANSYS software numerically solves the equilibrium equations to determine the full stress state, from which the maximum principal stress is extracted and subsequently compared to the temperature-dependent flexural strength (*σ_f_ (T)*) for failure–resilience assessments. In this study, the temperature-dependent flexural strength (*σ_f_ (T)*) is used as the material’s strength limit. As outlined in Table 1, the room-temperature flexural strength (*σ_f_*_0_) of the fully dense composite is 357.78 MPa.

#### 2.3.2. Meshing and Convergence Study

To ensure mesh independence and numerical accuracy, six mesh configurations were developed across both the simple and complex geometrical models. These included baseline coarse, uniform fine, and locally refined custom meshes focused on the throat region. The nomenclature and characteristics of each mesh are summarized in Table 2. The mesh independence study used the most structurally critical moment, i.e., the time step corresponding to the maximum observed thruster stress. This peak occurs early in the thrusting phase (~2.16 s), where thermal gradients and stress concentrations reach their extreme values.

**Figure 2:**
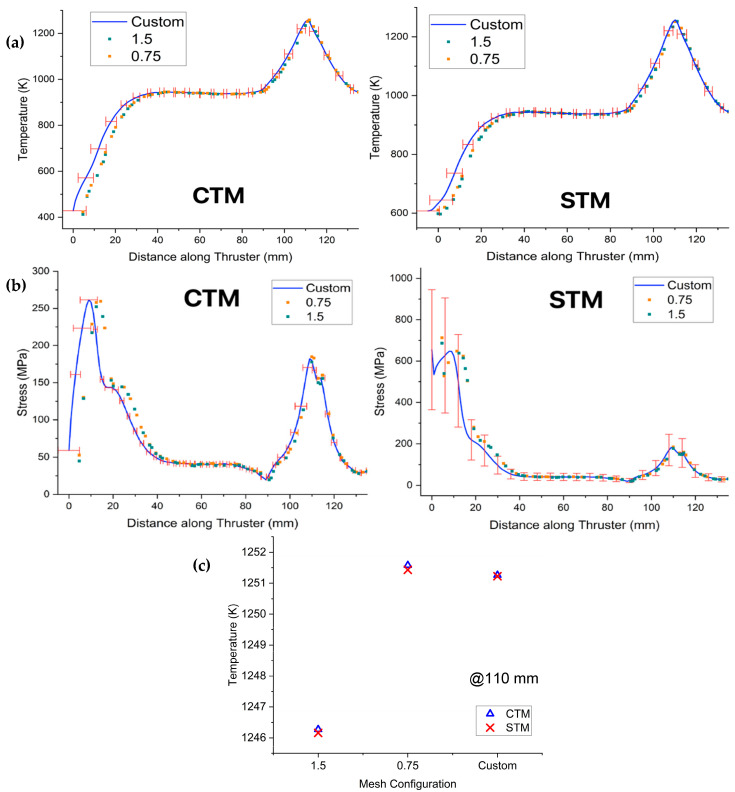
Mesh comparison for temperature (**a**) and stress (**b**) on the inner surface of both the thruster configurations at the time step of maximum nozzle stress. Temperature convergence at the 110 mm axial position (**c**) for the STM and CTM across various mesh configurations.

Figure 2a,b illustrates the temperature and stress data comparisons, respectively, across different mesh resolutions for both the STM and CTM. This figure shows the mean standard deviation from the Custom mesh with the 0.75 and 1.5 mesh sizes. The finer meshes (STM0.75 and CTM0.75) closely follow the refined custom mesh results (STM_custom_ and CTM_custom_), with deviation in maximum stress values remaining within 2.5%. This confirms that mesh convergence has been achieved.

Figure 2c focuses on a detailed temperature convergence analysis at the critical 110 mm axial position. This plot shows the predicted temperature values for both the CTM and STM across the 1.5 mm, 0.75 mm, and custom mesh configurations. As depicted, the temperature predictions for both models exhibit minimal variation between the 0.75 mm and custom mesh configurations, further validating the mesh independence at this specific point. For instance, the temperature difference for the CTM between the 0.75 mm and custom meshes is only approximately 0.31 K, and similarly, for the STM, it is about 0.28 K.

Quantitative metrics for stress and temperature convergence are shown in Table 3. The custom mesh (CTM_custom_) was selected as the final working mesh due to its smooth stress response in high-gradient regions while maintaining computational feasibility. The custom mesh was selected for its ability to maintain high fidelity in stress-critical regions while ensuring consistent element quality across all wall thickness configurations. It incorporates localized refinement from the chamber convergence through the divergent section, with particular focus on the throat, where peak stresses and sharp gradients are expected. This approach enables smoother, non-erratic stress plots and allows direct control over edge sizing, ensuring that varying thickness geometries are meshed uniformly and comparably.

## 3. Results

### 3.1. Model Fidelity Comparison: Simple vs. Complex Thruster Geometry

**Figure 3:**
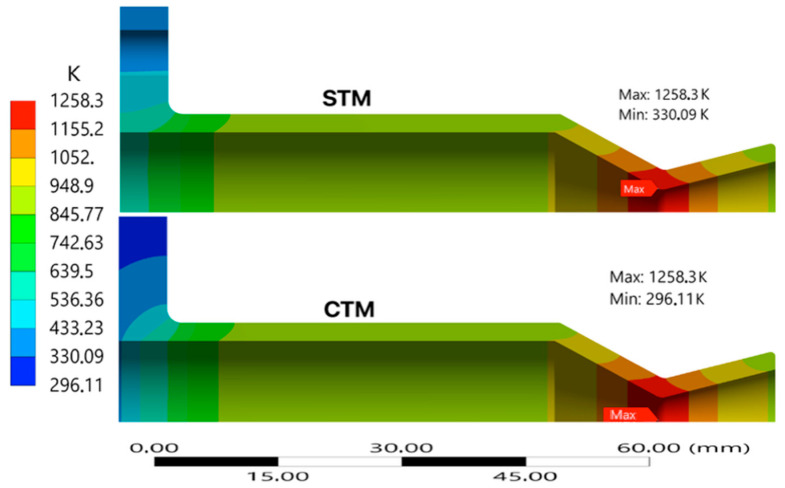
Comparison of transient thermal contour maps for thruster configurations.

**Figure 4:**
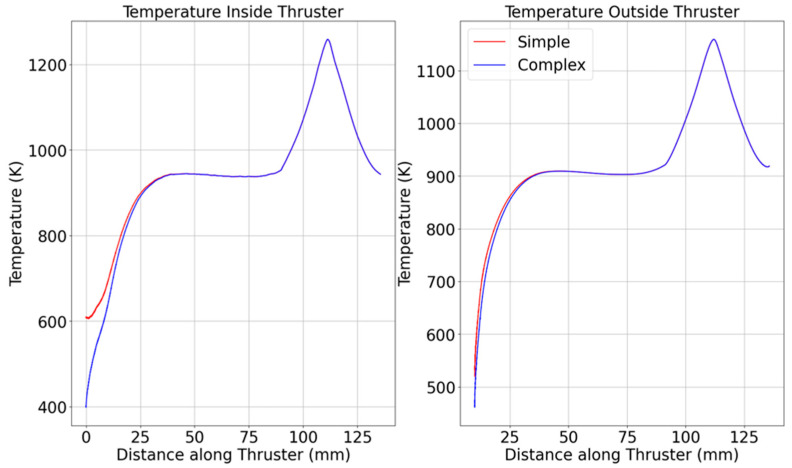
Axial temperature profiles (wall centerline) for STM vs. CTM at t = 2.03 s.

To ensure accurate stress and thermal predictions for sustainable thruster design, two geometric configurations were evaluated with a uniform wall thickness of 4 mm for a simplified axisymmetric STM and a more detailed full-featured CTM geometry. Figure 3 and Figure 4 compare the transient thermal behavior of the STM and CTM using wall temperature contours and axial temperature distribution, respectively. In the STM configuration, a maximum temperature of 1258.3 K is reached at the convergent section of the throat. The CTM closely follows the STM and shows higher peak temperatures near the throat. The better-defined gradients along the thruster profile are attributed to geometric transitions that intensify local convective heating. The maximum temperature in the CTM shows a difference of 0.0011% compared to the simple model.

As seen in Figure 4, the STM underpredicts wall heating effects, especially at the convergent-throat interface. The temperatures largely coincide for the CTM and STM, except near the beginning and closer to the back plate. Specifically, for the inner and outer measurements, the temperatures of the CTM are within 2% of those in the STM after 18.7 mm for both inside and outside temperature measurements. As expected, the temperature in the CTM is slightly lower, most likely due to the additional material, which conducts heat, causing the temperature at the probed areas to decrease.

**Figure 5:**
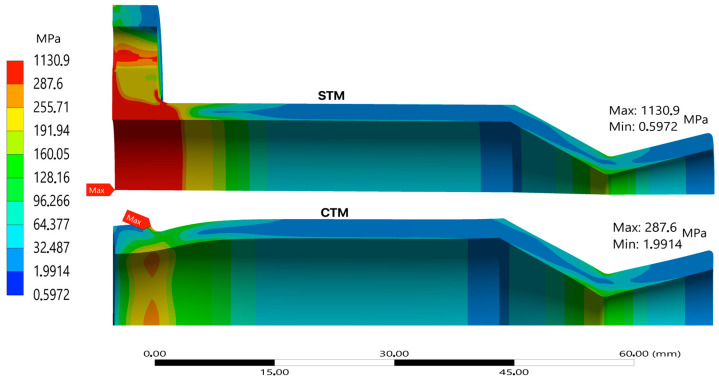
Maximum principal stress contours for both the thruster configurations.

Structural performance diverges significantly. Figure 5 displays a comparison of the static structural stress distributions under equivalent loading in the STM and CTM. While the STM captures broad stress zones, the CTM reveals higher local stress concentrations around geometric transitions, especially at the fillet near the chamber-throat junction and outer flange edges. In the STM, the stresses near the support are significantly higher (expected due to the fixed support constraints) than in other parts of the thruster, including the expected peak stress in the nozzle’s convergent sections near the throat. In the case of the CTM, the high stresses seen near the flange have been greatly reduced; so much so, that they are now below the fracture strength of the UHTCMC material. Similarly to the STM, the stresses near the flange are still greater than those experienced at the nozzle.

**Figure 6:**
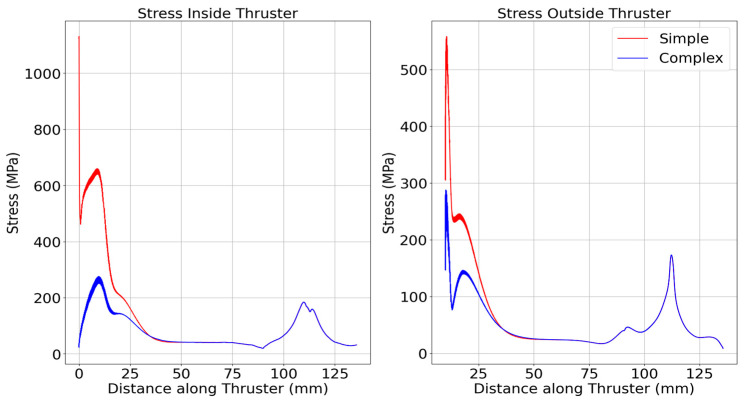
Axial stress distribution along the thruster length for STM vs. CTM.

Figure 6 further confirms this, showing elevated hoop stress on the CTM outer wall, highlighting its sensitivity to curvature and load-path detail. Notably, the largest stress differences between the STM and CTM occur near the flange on the thruster’s interior. Beyond 34.0 mm (inside) and 34.8 mm (outside), this difference drops to less than 2%, indicating that flange stress concentrations have minimal impact beyond a certain point. The CTM reveals two distinct stress peaks: one in the nozzle’s convergent section and another at the 3 mm curved flange section. This additional detail was missed in the STM, though a small peak around 11 mm on the inside stress curve hinted at surface curvature stress concentrations.

**Figure 7:**
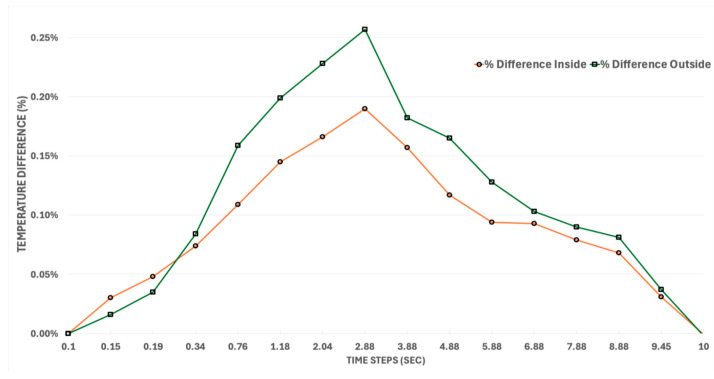
Inside and outside wall temperature difference for the STM and CTM configurations over time.

The temporal evolution of temperature and stress also differs meaningfully between the two models. Figure 7 shows the percentage difference between the STM and CTM for both inner and outer wall temperatures, providing insight into the accuracy and limitations of using simplified thermal assumptions in thruster analysis. The temperature increases rapidly at the beginning, and the rate of increase starts to decrease as time progresses. A low percentage difference across all time steps suggests that the STM is a reasonable approximation for preliminary design. However, noticeable differences during transient heating phases highlight the importance of capturing detailed thermal interactions, such as localized conduction, realistic boundary conditions, or variable material properties, which are typically only present in the CTM.

**Figure 8:**
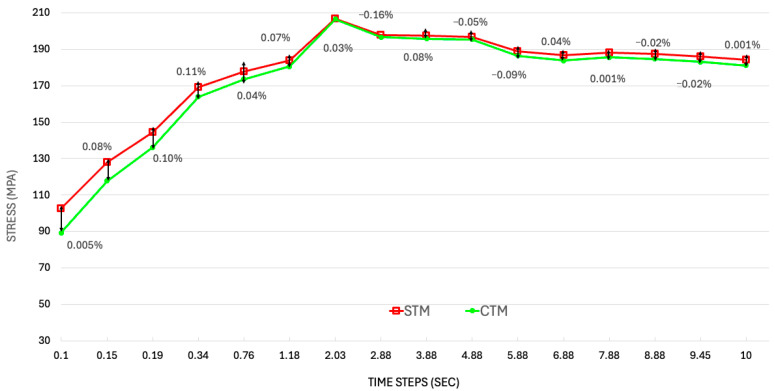
Maximum principal stress over time along the thruster configurations. (The black arrows simply highlight the difference in stress between STM and CTM values).

Figure 8 illustrates the percentage difference between the STM and CTM stress profiles over time, providing a clear understanding of how structural stress predictions vary between the two modeling approaches. The consistently small percentage differences, mostly within ±0.1%, indicate a strong agreement between the STM and CTM throughout the operation period, suggesting that the simplified model captures the essential stress behavior with reasonable accuracy. This reinforces the reliability of using the STM for early stage structural assessments where computational efficiency is prioritized. However, the slight deviations at specific time steps, including both positive and negative values, point to localized differences in how each model handles thermal gradients, boundary conditions, and material interactions under load. These subtle discrepancies, although minor, highlight that the CTM may still offer valuable refinements in high-fidelity analyses where precise stress margins and critical failure–resilience assessments are essential.

**Figure 9:**
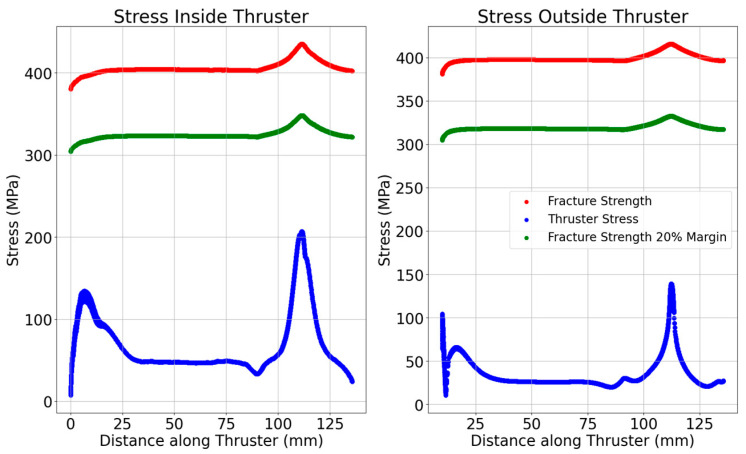
Stress margin evaluation at 2.03 s for the CTM.

The peak stress in the CTM occurs at 2.03 s. This early and sharp stress rise is a direct consequence of localized thermal buildup and the presence of stress-raising features such as sharp transitions and reduced fillet radii. Figure 9 shows that the stresses in the thruster do not exceed the fracture strength of the material. The thruster has a 4 mm thickness near the flange, which prompted further investigation to determine if thinner configurations could exceed the material’s fracture strength. Preliminary simulations revealed that thrusters with less than 4 mm thickness near the flange would indeed surpass the fracture strength. This finding is crucial, as it limits the thruster’s design options, requiring a minimum 4 mm thickness at the chamber’s start to ensure structural integrity.

Based on these comparisons, it is evident that the CTM offers superior predictive resolution for both temperature and stress behavior. The STM, while computationally efficient, underestimates peak thermal gradients and fails to capture stress concentrations arising from geometry-specific features. As such, the remainder of this study, including wall thickness optimization and degradation evaluation, is carried out using the CTM as the baseline configuration.

### 3.2. Wall Thickness Optimization

Following the selection of the CTM for all further evaluations, a detailed study was carried out to investigate how varying wall thicknesses affect the structural performance and thermal response of the thruster. Given that high stress concentrations are typically observed near the flange, the chamber wall adjacent to the flange was fixed at 4 mm in all configurations to maintain mechanical integrity during operation. The remaining thruster sections, specifically the convergent and divergent regions, were varied in thickness to assess their influence on the system’s overall behavior.

The thicknesses of these two regions were chosen from discrete values of 2 mm, 3 mm, and 4 mm, resulting in a total of nine unique combinations. Notably, the convergent section thickness was kept consistent along its length by ensuring that the chamber wall slightly upstream of the convergent profile matched its value. This approach maintains a continuous structural profile without abrupt wall transitions in the converging zone. The nine configurations analyzed are listed in Table 4.

**Figure 10:**
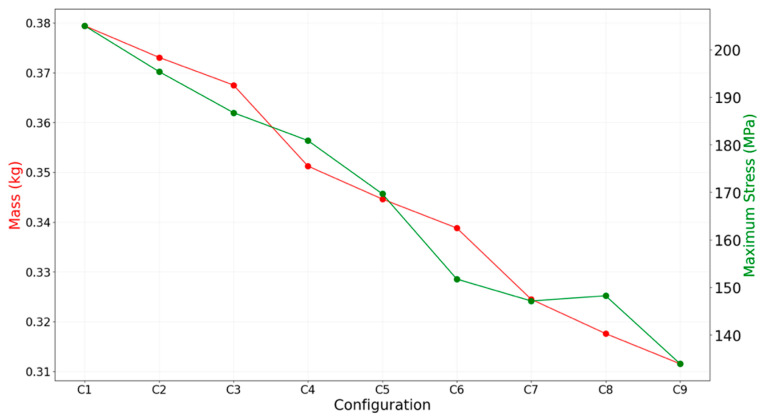
Mass and maximum principal stress for configurations C1–C9.

Figure 10 presents the mass and maximum principal stress values for each of the nine configurations. A clear inverse relationship between mass and stress is observed. As the nozzle becomes thinner, particularly in the divergent and convergent sections, the thermal mass decreases, reducing the through-thickness temperature gradient and thereby lowering thermal stress. This trend aligns with observations from prior work in thermo-structural analysis of nozzles in aero-engine applications, which also showed decreasing stress levels with reducing wall thicknesses [17].

**Figure 11:**
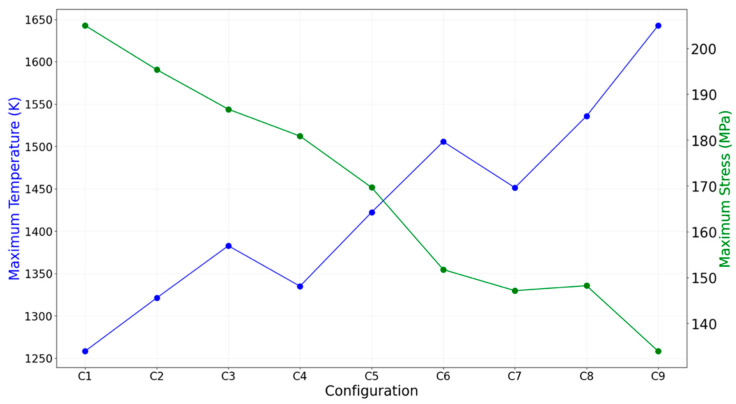
Maximum temperature vs. maximum principal stress for all configurations.

Figure 11 shows the relationship between maximum temperature and stress across the configurations. Thinner thrusters tend to heat up more rapidly due to their lower thermal inertia, which raises the overall wall temperature despite lowering structural stress. This presents a fundamental trade-off: reduced thickness results in lower stress but higher surface temperatures. However, since the UHTCMC material used in this study is capable of withstanding temperatures up to ~2200 K without degradation [10,11,16], thermal limits do not constrain the design space.

**Figure 12:**
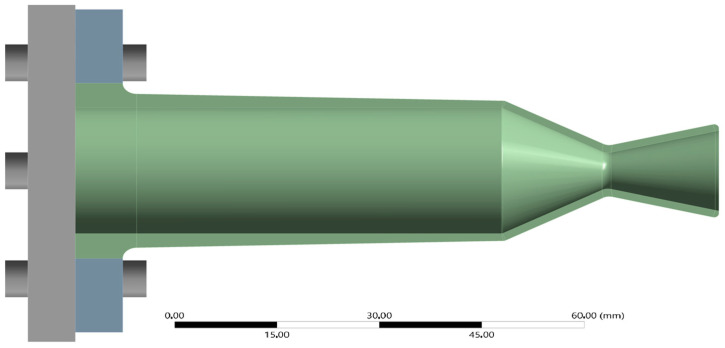
Side view of optimal configuration (C9), showing continuous taper from flange to exit.

From a structural standpoint, stress and weight remain the critical parameters. Configuration C9 with a 2 mm thickness in both the convergent and divergent sections demonstrate the lowest mass (0.31 kg) and the lowest peak stress. This makes it the optimal configuration for achieving a lightweight, high-durability nozzle suited for green bipropellant systems. The geometry of this design is visualized in Figure 12. It includes a gradual taper from 4 mm at the flange to 2 mm at the convergent zone, ensuring smooth load transfer. While this introduces additional geometric complexity, especially for additive manufacturing, it represents a compelling balance between structural integrity and material economy. All further thermal, structural, and degradation analyses presented in the following sections are based exclusively on this optimized configuration (C9).

While the optimized geometries offer significant performance advantages, their practical realization, particularly through emerging additive manufacturing (AM) techniques, presents distinct challenges. Current 3D printing technologies for UHTCs or UHTCMCs are still under extensive research, and several anticipated difficulties remain unaddressed in the literature. These include the complex behavior of the material as it exits a nozzle in liquid form and solidifies into a specific shape, the need for highly controlled pressure and temperature environments during the curing process, and the intricate control required over fiber orientation to achieve desired microstructures. Furthermore, accurately capturing detailed features such as the 3 mm rounded edges of the nozzle can be challenging for current AM systems, and preventing defects like air gaps in shallow, tapered surfaces, often seen even with common materials like PLA, is crucial for UHTCMCs. Advanced approaches, such as 6-axis 3D printing, show promise in addressing some of these complexities by offering capabilities to simulate randomly oriented chopped fibers through smart programming and improve geometric fidelity.

### 3.3. Transient Thermal Response and Heat Transport

**Figure 13:**
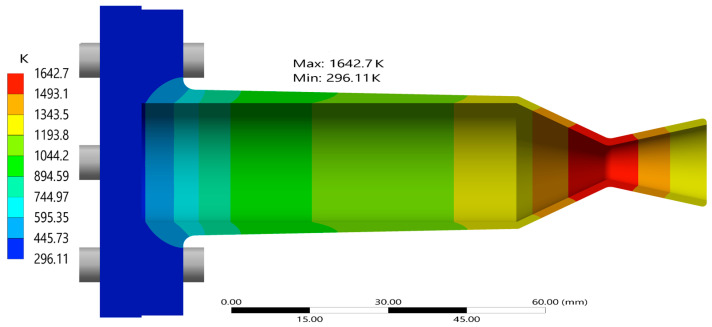
Temperature contour of the inner surface of the thruster at 10 s.

The thermal behavior of the optimized nozzle configuration (C9) was analyzed by examining the temperature distribution across both the inner and outer walls of the thruster. The geometry incorporates a gradual thickness reduction, and this tapering has a significant influence on the way heat is absorbed and conducted through the nozzle structure. Figure 13 illustrates the contour of temperature distribution on the thruster wall, the surface directly exposed to the hot combustion gases. The higher temperatures are concentrated on the throat region, where convection coefficients and near-wall temperatures are highest. Further away from the throat at the nozzle exit, the temperatures drop rapidly from the peak of 1642.7 K, at the end of the operation time, 10 s.

**Figure 14:**
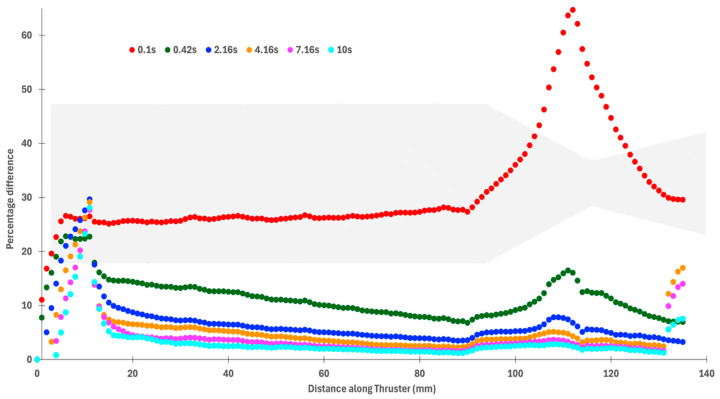
Percentage difference in inner and outer wall temperature for the thruster over multiple time steps.

Figure 14 presents the percentage difference in the inner and outer wall temperature for the thruster over the whole run duration of 10 s. The chamber wall acts as a thermal conduit, transferring heat from the high-temperature regions in zone C (the nozzle) towards the thicker upstream walls near the flange. Because heat conduction is influenced by the local wall thickness [7], the temperature profile becomes steeper where the geometry transitions from thin to thick. This is particularly evident near the junction between the convergent and the chamber, where the wall thickness increases sharply. The sharp change in slope is likely due to the greater thermal inertia of the flange, which slows down heat propagation in that region. A small kink around the 10 mm axial position suggests a bottleneck in heat transfer as energy begins to spread laterally into the larger bulk mass of the flange. The percentage difference between the inside and outside temperature is huge at 0.1 s, as temperatures on the outer wall are much lower than on the inner surface due to conductive losses through the material.

**Figure 15:**
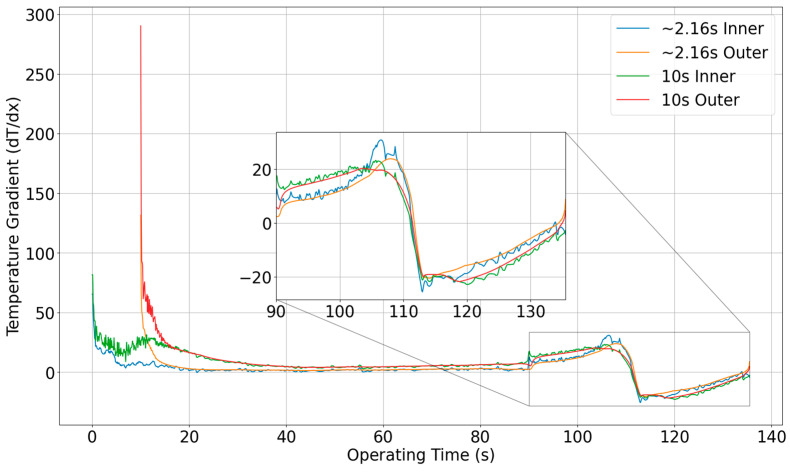
Temperature gradient contour of inner and outer surfaces at 2.16 s and 10 s.

A more detailed understanding of this transient thermal behavior is captured in Figure 15, which shows the temperature gradient contours for both the inner and outer surfaces at two different time steps: 2.16 s (the time of peak structural stress) and 10 s (the end of the operational pulse). At 2.16 s, the temperature gradient near the flange is relatively low, reflecting the fact that thermal energy has not yet fully propagated through the thicker upstream sections. In contrast, by the 10 s, the gradient becomes much steeper, particularly in the convergent section, where the thermal wave has advanced. This is consistent with the observations from the previous figures that the combination of thinner wall sections and sustained heat input leads to an accelerated thermal response.

Interestingly, the zoomed-in view of the throat region indicates that the peak temperature gradient in the converging section is actually higher at 2.16 s than at 10 s. This reflects a rapid, localized accumulation of thermal energy early in the pulse, before it diffuses downstream. Over time, as more energy is absorbed by the system, the peak gradient relaxes slightly, though the average gradient across the entire nozzle increases. These observations confirm the physical expectation that thinner sections heat more quickly, but the thicker chamber and flange regions act as thermal buffers, absorbing and redistributing energy more slowly.

### 3.4. Stress Evolution and Structural Response

Understanding the development of thermal-mechanical stress throughout the thruster during operation is essential to assessing structural integrity, particularly under transient loading. In this section, the time-dependent stress evolution is analyzed using full-field contour maps, gradient plots, and axial deformation data for the optimized configuration C9. All observations focus on transient loading effects over a 10 s operation cycle.

**Figure 16:**
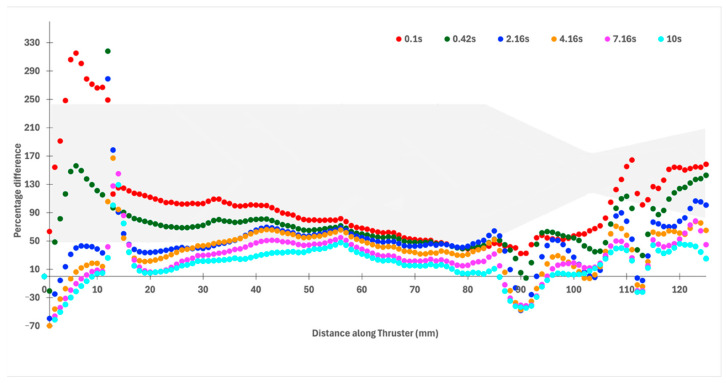
Percentage difference in inner and outer wall stress profiles for the thruster over multiple time steps.

Figure 16 presents the percentage difference between the inner and outer wall stress profiles throughout the entire thruster as a function of time. This plot aggregates the maximum stress across zones A, B, and C at each timestep. The highest stress in the nozzle occurs at 2.16 s; however, flange stresses begin to exceed nozzle stresses shortly thereafter. Notably, the inner flange wall already exhibits higher stress than the nozzle at 1.84 s. The outer surface of the flange, specifically around the 3 mm curvature, ultimately bears the highest stress during the burn. This localized peak suggests that the curvature geometry amplifies surface stress near the flange attachment. This is the reason behind the large percentage difference observed up to 18 mm of the thruster at the initial time steps (0.1–2.16 s).

**Figure 17:**
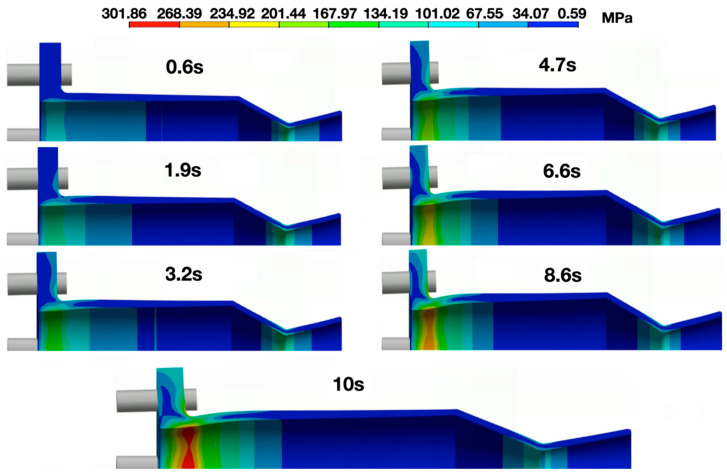
Stress progression contours from t = 0 to 10 s on the thruster.

The rapid progression of stress through the nozzle and flange regions is further illustrated in Figure 17. These contour snapshots at multiple time steps visually confirm that the stress in zone C rises sharply in the first few seconds of operation but is eventually overtaken by the flange region as the structure continues to heat.

**Figure 18:**
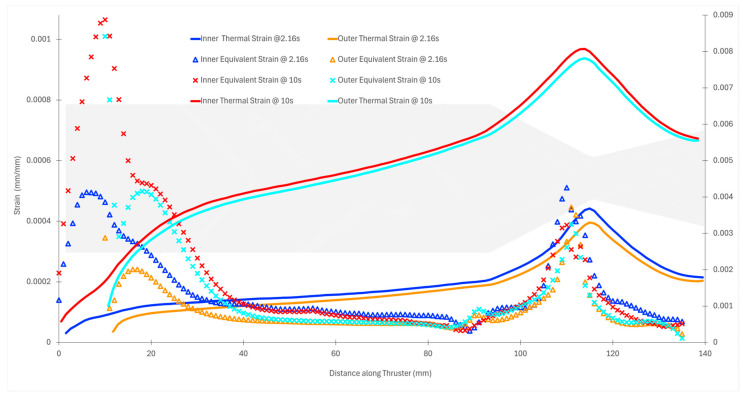
Strain distribution along the thruster at peak and whole run duration.

Strain distributions offer additional insight into structural behavior under combined thermal and pressure loads. At the peak stress time (2.16 s), thermal strains are highest near the throat, as shown in Figure 18. While flange stress may dominate due to geometry, the nozzle throat remains the region of highest thermal-induced material elongation. Over longer durations, strain intensifies due to increasing temperature and concurrent degradation of the material’s Young’s modulus. As seen in Figure 18, by 10 s, the thermal strain in the nozzle section becomes nearly 16 times higher than that in the flange. In comparison, the flange strain increases by a factor of approximately 3.5. This disparity arises from the fact that the modulus of elasticity degrades faster at high temperatures than the rate of thermal strain growth, resulting in lower net stress but greater deformation. This confirms that while stress values may fall at longer durations, strain continues to accumulate, particularly in thin-walled, high-temperature regions.

**Figure 19:**
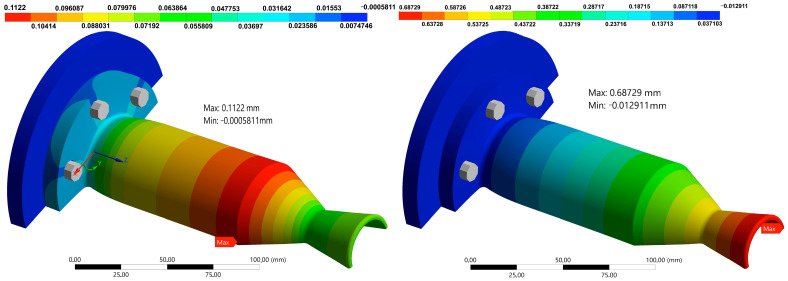
Radial (**left**) and Axial (**right**) deformations on the optimum thruster design at 10 s.

Lastly, the overall structural deformation of the thruster is depicted in Figure 19. The total axial expansion at the nozzle exit reaches 0.687 mm at 10 s, approximately 0.51% of the total thruster length (135.72 mm). Maximum radial deformation occurs at the start of the convergent section, corresponding to the region where chamber pressure is highest and the wall begins to taper. In the transverse direction, deflection near the curved flange section also rises slightly due to the pinching effect at the mounting curvature, consistent with earlier axial stress profiles.

These structural trends confirm that thermal strain is the dominant source of deformation during operation, even though thermal stress reduces as the modulus degrades. The challenge remains in separating how much of the total strain results from free thermal expansion versus constrained deformation, which contributes to internal stress buildup. However, based on current simulations, it is clear that thermal stress remains the leading cause of structural loading in the early burn period, while thermal strain governs long-term deformation behavior.

### 3.5. Damage and Degradation Considerations

While absolute stress values are crucial in evaluating structural response, long-term durability and failure–resilience assessment is better assessed through stress margin analysis, that is, the difference between the applied principal stress and the flexural strength of the material at operating temperature. Because the flexural strength of UHTCMC degrades with increasing temperature, the locations most susceptible to failure may not necessarily be those with the highest absolute stress, but rather those with the smallest stress margin at a given time.

**Figure 20:**
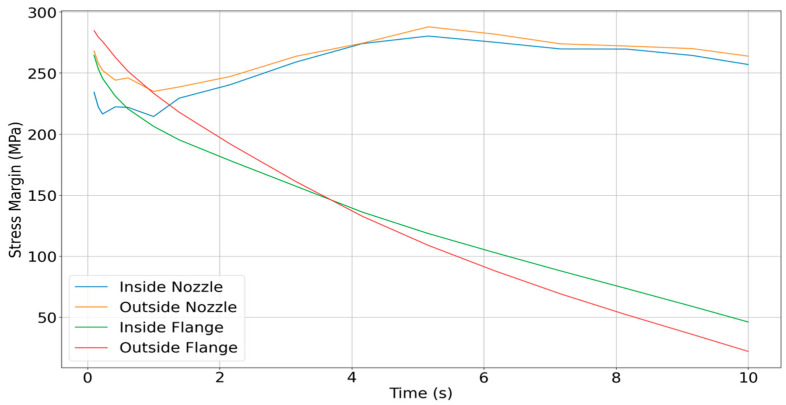
Stress margin evolution at four critical thruster locations.

Figure 20 presents the calculated stress margins at four critical regions of the thruster. These include (i) the inner nozzle wall, (ii) the inner wall near the flange, (iii) the outer flange curvature, and (iv) the outer nozzle wall. Each of these zones experiences different heating and mechanical loading profiles over time, and thus, they transition in failure risk as the burn progresses. Importantly, the location of potential failure shifts during the thrusting cycle. Although the maximum nozzle stress occurs at 2.16 s, the flange region becomes structurally critical far earlier, as early as 0.59 s, when evaluated in terms of stress margin. This is due to the relatively rapid rise in temperature at the flange, which causes a sharp drop in local flexural strength.

Based on this analysis, the most critical degradation locations change as a function of thrust duration:

- 0–0.59 s: The most likely failure point is the convergent section near the throat. Though absolute stress is still ramping up, the thin-walled region near the high-temperature throat is vulnerable to erosion and micro-cracking. Long-term cyclic operation in this regime could result in gradual material degradation.
- 0.59–3.68 s: During this period, failure risk shifts to the inner surface near the flange, particularly around the 10 mm axial location. This area experiences intermediate temperatures but higher cumulative thermal strain over time. Cracks may develop from repeated thermal cycling, especially if operation durations in this range occur frequently.
- 3.68–10 s: For longer-duration burns, the most critical damage is likely to occur at the outer bend where the flange joins the chamber body. This region exhibits the lowest stress margin during sustained operation and is vulnerable to the propagation of small cracks, whether introduced during manufacturing or accumulated through cyclic thermal loading.

**Figure 21:**
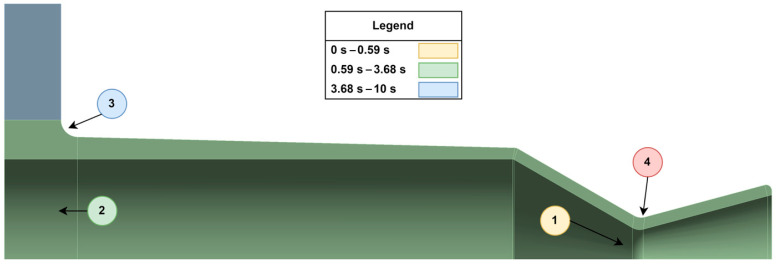
Degradation risk map based on thrust duration. (The red-colored number 4 represents a non-critical region that, under the evaluated operating conditions, will consistently remain outside the locations of initial fracture and is thus, not included in the legend).

Figure 21 illustrates these degradation zones graphically, highlighting which areas of the nozzle are most likely to fail as a function of operating time. If the thruster is operated consistently within a specific burn duration window throughout its lifetime, Figure 21 can be used to anticipate the most probable fracture origin. In all cases, the analysis reinforces the importance of designing for the intended duty cycle. Given that small upper-stage thrusters often operate in short bursts under 3 s, the nozzle throat and inner flange wall should be prioritized for structural reinforcement and life prediction modeling.

It is also important to note that all degradation predictions in this study are based on thermal stress margins under hot-fire conditions without any active or passive cooling mechanisms. The inner and outer wall temperatures, as well as the resulting stress margins, reflect the most conservative thermal load scenario that is a fully insulated structure subjected to continuous heating over a 10 s burn. In real-world applications, radiative or conductive cooling, even modest in nature, would significantly reduce the thermal gradient across the nozzle wall, thereby increasing the local material strength and improving the stress margin in all regions. As such, if cooling were incorporated into the design or operation, the expected damage onset and material degradation would be further delayed, likely extending well beyond the 10 s operational window considered here. This reinforces the structural viability of the optimized thruster configuration under actual mission conditions.

## 4. Conclusions

A fully coupled transient thermo-structural analysis was performed to investigate the performance of a UHTCMC thruster for green bipropellant thrusters. Comparison between an STM and a detailed CTM showed that the CTM better captured thermal gradients and stress concentrations, particularly near the flange and convergent sections. Overall, nine wall thickness configurations were analyzed, leading to the following major conclusion from this study:

1. The optimized configuration (C9) with 2 mm tapering in both convergent and divergent sections offered the lowest mass (0.31 kg) and stress while maintaining structural integrity.
2. Thermal analysis revealed that peak gradients occur near the throat early in the burn (around 2.16 s), with thermal strain becoming dominant in later stages due to degradation in material stiffness.
3. Stress margin-based damage evaluation identified three critical failure zones based on thrust duration: (i) throat for <0.59 s, (ii) inner flange for 0.59–3.68 s, and (iii) outer flange transition for >3.68 s.

The analysis was performed without any cooling. Under realistic radiative or conductive cooling, stress margins would improve further, extending operational safety beyond 10 s. This study establishes a scalable methodology for sustainable thruster design using UHTCMCs, with direct implications for reusable, green propulsion systems in space applications. While this study provides robust numerical insights into the design optimization of UHTCMC thruster, the absence of direct experimental validation for the studied specific design under the simulated transient conditions is acknowledged. Future work will focus on conducting experimental campaigns to corroborate these numerical models, which is crucial for full design qualification and further enhancing the credibility of our findings.

## Figures and Tables

**Table 1:** Temperature-dependent material properties of ZrB_2_–SiC–C fiber composite.

Temperature (K)	Young’s Modulus (GPa)	CTE (×10^−6^ K^−1^)	Thermal Conductivity (W/(m·K))	Specific Heat (J/(kg·K))	Flexural Strength (MPa)
RT (300)	267.47	4.00	38.18	554.78	357.78
500	261.38	5.21	48.69	910.40	–
1000	246.34	5.80	47.09	1053.25	509.72
1500	236.65	6.06	54.77	1156.08	457.10
2000	204.41	–	54.81	1209.78	–
2500	180.52	6.42	59.74	1237.84	70.10

**Table 2:** Mesh resolution configurations and identifiers.

Mesh ID	Throat Element Size (mm)	Approx. No. of Elements	Description
STM1.5	1.5	460,000	Coarse baseline mesh
STM0.75	0.75	910,000	Uniform fine mesh
STM**_custom_**	0.75 (with local refinement)	1,200,000	Custom mesh focused on throat
CTM1.5	1.5	650,000	Coarse full geometry mesh
CTM0.75	0.75	1,380,000	Uniform fine mesh
CTM**_custom_**	0.75 (with local refinement)	1,820,000	Custom mesh focused on throat

**Table 3:** Percentage differences in maximum values (temperature, T and stress, S) at the time step of peak stress in the nozzle compared to the custom mesh complex model results.

Parameters	STM			CTM		
	1.5	0.75	Custom	1.5	0.75	Custom
T in	0.24	0.28	0.17	0.11	0.1	0.09
T out	0.09	0.33	0.23	0.11	0.1	0.08
S in	41.3	90.07	112.04	0.71	1.64	0.7
S out	9.84	12.78	15.22	6.71	5.53	3.11

**Table 4:** Thruster configurations based on wall thickness variations.

Configuration	Convergent Section Thickness (mm)	Divergent Section Thickness (mm)
C1	4	4
C2	4	3
C3	4	2
C4	3	4
C5	3	3
C6	3	2
C7	2	4
C8	2	3
C9	2	2

## Data Availability

The original contributions presented in this study are included in the article. Further inquiries can be directed to the corresponding author.
